# Evaluation of CNPase and TGFβ1/Smad Signalling Pathway Molecule Expression in Sinus Epithelial Tissues of Patients with Chronic Rhinosinusitis with (CRSwNP) and without Nasal Polyps (CRSsNP)

**DOI:** 10.3390/jpm14090894

**Published:** 2024-08-23

**Authors:** Katarzyna Piszczatowska, Katarzyna Czerwaty, Karolina Dżaman, Natalia Jermakow, Jacek Brzost, Ireneusz Kantor, Nils Ludwig, Mirosław J. Szczepański

**Affiliations:** 1 Department of Biochemistry, Medical University of Warsaw, 02-091 Warsaw, Poland; kpiszczatowska@wum.edu.pl (K.P.); nils.ludwig@fau.de (N.L.); 2 Department of Otolaryngology, The Medical Centre of Postgraduate Education, 01-813 Warsaw, Poland; katarzynaczerwaty@gmail.com (K.C.); kfrydel@poczta.onet.pl (K.D.); ireneusz.kantor@gmail.com (I.K.); 3 Department of Hyperbaric Medicine, Military Institute of Medicine—National Research Institute, 04-141 Warsaw, Poland; njermakow@gmail.com; 4 The Children’s Memorial Health Institute, 04-730 Warsaw, Poland; jbrz102018@gmail.com

**Keywords:** chronic rhinosinusitis, TGFβ1, Smad2, pSmad3, CNPase, adenosine, airway epithelium

## Abstract

Chronic rhinosinusitis with and without nasal polyps (CRSwNP and CRSsNP, respectively) is a chronic inflammatory disease affecting almost 5 to 12% of the population and exhibiting high recurrence rates after functional endoscopic sinus surgery (FESS). TGFβ1-related pathways contribute to tissue remodelling, which is one of the key aspects of CRS pathogenesis. Additionally, adenosine signalling participates in inflammatory processes, and CNPase was shown to elevate adenosine levels by metabolizing cyclic monophosphates. Thus, the aim of this study was to assess the expression levels of Smad2, pSmad3, TGFβ1, and CNPase protein via immunohistochemistry in sinus epithelial tissues from patients with CRSwNP (*n* = 20), CRSsNP (*n* = 23), and non-CRS patients (*n* = 8). The expression of Smad2, pSmad3, TGFβ1, and CNPase was observed in the sinus epithelium and subepithelial area of all three groups of patients, and their expression correlated with several clinical symptoms of CRS. Smad2 expression was increased in CRSsNP patients compared to CRSwNP patients and controls (*p* = 0.001 and *p* < 0.001, respectively), pSmad3 expression was elevated in CRSwNP patients compared to controls (*p* = 0.007), TGFβ1 expression was elevated in CRSwNP patients compared to controls (*p* = 0.009), and CNPase was decreased in CRSsNP patients compared to controls (*p* = 0.03). To the best of our knowledge, we are the first to demonstrate CNPase expression in the upper airway epithelium of CRSwNP, CRSsNP, and non-CRS patients and point out a putative synergy between CNPase and TGFβ1/Smad signalling in CRS pathogenesis that emerges as a novel still undiscovered aspect of CRS pathogenesis; further studies are needed to explore its function in the course of the chronic inflammation of the upper airways.

## 1. Introduction

Chronic rhinosinusitis (CRS) is a multifactorial inflammatory disease with a prevalence of 5 to 12% in the global population and is further clinically classified into CRS with nasal polyps (CRSwNP) and without nasal polyps (CRSsNP) [1]. CRS is characterised by an ambiguous molecular background and high recurrence rates, particularly in patients with CRSwNP [2,3,4,5,6]. Interestingly, recent evidence suggests that familial predisposition is a relevant factor in the pathogenesis of CRSwNP [7,8]. One of the key aspects involved in CRS pathogenesis is airway tissue remodelling [9,10,11,12,13,14,15]. The interior of the nose and paranasal sinuses has a surface area from 100 to 200 cm^2^ and is covered with pseudostratified epithelium (upper airway epithelium; UAE) [16], which is crucial for maintaining the proper functionality of the sinuses. Persistent inflammation triggers AE remodelling, specifically epithelial-to-mesenchymal transition (EMT) [14]. A growing body of evidence suggests an involvement of the TGFβ/Smad pathway in CRS pathogenesis. TGFβ1 mediates microenvironmental context-dependent processes, including immunoregulation, fibrosis, tissue remodelling, angiogenesis, metastasis, tumour progression, myofibroblast differentiation, and pro- or anti-inflammatory capacities [10,11,17,18,19,20,21,22,23,24,25,26,27]. TGFβ1 acts through both Smad-dependent and Smad-independent signalling pathways. In the canonical Smad-dependent pathway, TGFβ1 actives TGFβ1 type II and type I receptors, leading to the subsequent phosphorylation of Smad2 and Smad3 (receptor-activated Smads). The Smad 2/3 complex binds to Smad4 (Co-Smad) and translocates to the nucleus, where it regulates the expression of genes involved in processes such as proliferation, differentiation, immunoregulation, and tissue remodelling [19,28]. The detailed role of Smad2 and Smad3 in TGFβ1 signal transduction is currently under investigation [29]. Recently, adenosine (ADO) and cAMP-related signalling emerged in the context of the pathogenesis of chronic upper airway inflammatory diseases [30]. ADO is an endogenous purine nucleoside with a wide range of intra- and extracellular activities in both physiological and pathological conditions and can exert pro- or anti-inflammatory activity. The effects of ADO depend on its concentration, microenvironmental conditions, duration of action, and the types of receptors activated on the surface of target cells [30]. In addition to ATP hydrolysis, which is a well-known source of ADO, mRNA breakdown during tissue injury and cellular damage has been identified as an alternative source [31]. 2′,3′-cAMP derived from mRNA is converted via CNPase (2′,3′-cyclic nucleotide 3′- phosphodiesterase; CNP) to 2′-AMP, which is subsequently transformed by ectonucleotidases into extracellular ADO [32,33]. CNPase is primarily expressed in myelinated nerve cells, where it supports nerve myelination and regulates microtubule activity. However, it is also expressed by non-myelinated cells such as olfactory ensheathing cells [34] and lens epithelium cells [35]. It has been shown that the TGFβ-dependent expression of CD73 is involved in the generation of ADO by regulatory T cells (Tregs), CD8+ T cells, dendritic cells, and macrophages [36]. Additionally, increased cAMP levels have been found to alter the TGFβ- and Smad-induced expression of extracellular matrix (ECM) components and fibroblast activity [37]. Furthermore, CNPase has been shown to contribute to epithelial remodelling via the TGFβ2–notch pathway, potentially playing a role in fibrosis and cataract development [35]. Additionally, ADO has been found to regulate fibroblast functions and activity, as well as the structure of the ECM through TGFβ signalling [38,39]. This regulation may play a crucial role in tissue remodelling related to CRS pathogenesis, particularly involving subepithelial nasal fibroblasts. In the nasal polyp tissue, the expression of both TGFβ1 and TGFβ2 isoforms has been demonstrated [40]. Another significant point is that CNPase associates with microtubules (MTs) [41] and interestingly, Smad2 and Smad3 proteins bind to MTs in the absence of TGFβ. Treatment with TGFβ leads to the dissociation of Smad 2 and Smad3 from MTs, followed by their phosphorylation, translocation to the nucleus, and activation of transcription [42]. Therefore, there may be a possible relationship between the expression of TGFβ, Smad2 and pSmad3, and CNPase in UAE and CRS pathogenesis, although this connection has yet to be clarified.

The molecular background of CRS remains elusive, necessitating extensive investigation to pave the way for more effective therapeutic and diagnostic solutions. The interplay between the TGFβ1, Smad, and CNPase pathways could represent a novel and yet undiscovered aspect of tissue remodelling that contributes to disease pathogenesis. Therefore, the aim of this study was to assess the expression of TGFβ1, Smad2, pSmad3, and CNPase in sinus epithelial tissues obtained from patients with CRSsNP, CRSwNP, or non-inflammatory controls, and to evaluate their mutual co-expression.

## 2. Materials and Methods

### 2.1. Patients

Samples of sinus tissue were obtained from 43 patients diagnosed with CRS, including 23 patients with CRSsNP and 20 patients with CRSwNP, who underwent functional endoscopic sinus surgery (FESS) for the first time in the Department of Otolaryngology, the Medical Centre of Postgraduate Education in Warsaw. The diagnosis of CRS was based on recommendations according to the European Position Paper on Chronic Rhinosinusitis (EPOS2020) [1]. The study included patients qualified for FESS for the first time due to insufficient disease control with pharmacological treatment. Exclusion criteria included previous sinus surgery, a diagnosis of cystic fibrosis, immunodeficiencies, smokers, systemic corticosteroids users, and systemic or local antibiotic therapy within four weeks prior to FESS. As controls, mucosal tissue samples were obtained from eight patients (NC; N = 8) undergoing surgery for non-CRS upper airway disorders, such as septoplasty or sleep apnoea syndrome. The mucosal samples in NC were taken from the lateral surface of the middle nasal concha, while in CRS patients, they were taken from the uncinate process. Additionally, normal retro auricular excess skin samples from ten patients operated on for middle ear cholesteatoma were used as positive staining control tissues for the tested antigens.

This study was approved by the Local Ethics Committee at the Medical Centre of Postgraduate Education (#50/PB/2019 to K.C. and #15/PB/2018 to I.K.). All subjects enrolled in the study completed the Sino-Nasal Outcome Test (SNOT-20) questionnaire [43] and signed informed consent forms. Patients were also interviewed for symptoms described in the EPOS 2020—main symptoms: nasal patency, mucus, sinus pain, and smell; additional symptoms: headache, fatigue, fetor ex ore, fever, toothache, cough, and earache. The questionnaire for patients based on the EPOS 2020 is included in Supplementary Table S1. The clinicopathologic characteristics of the CRS and control group patients included in this study are presented in Table 1.

### 2.2. Immunohistochemistry

Tissues were fixed in 10% buffered formalin solution, paraffin-embedded, and sectioned into 4 μM thick slices, and then mounted on the Adhesive Superfrost Plus Slides (Thermo Scientific, Waltham, MA, USA, J1800AMNZ). The following primary antibodies diluted in Antibody Diluent (Leica Biosystems Newcastle, Newcastle upon Tyne, UK, RE7133) were used for immunostaining: mouse monoclonal anti-human CNPase (1:200; Abcam, Cambridge, UK, ab6319), rabbit monoclonal anti-human anti-Smad2 (1:100; Cell Signalling, Danvers, MA, USA, 5339T), rabbit polyclonal anti-human anti-Phospho-Smad3 (1:400; St John’s Laboratory, London, UK, STJ114841), rabbit polyclonal anti-human anti-TGFβ1 (1:125, Abcam, Cambridge, UK, ab92486), or the appropriate isotype control IgG. After standard deparaffinisation, rehydration, and antigen retrieval in pH = 9 (Epitope Retrieval Solution, Novocastra, Leica Biosystems Newcastle, Newcastle upon Tyne, UK, RE7119) for 30 min in a water bath at 98 °C, sections were stained according to the manufacturer’s instructions for the Novolink Polymer Detection System (Leica Biosystems Newcastle, Newcastle upon Tyne, UK, RE7140-K). First, the activity of endogenous peroxidase was blocked with Novocastra^TM^ Peroxidase Block (Leica Biosystems Newcastle, Newcastle upon Tyne, UK, RE7101), and then Novocastra™ Protein Block was used to eliminate the non-specific binding of the primary antibody and polymer. Next, sections were incubated with the primary antibody or only with the Antibody Diluent (negative controls) for 75 min at room temperature (RT) in the moist chamber, followed by incubation with a secondary antibody—Novocastra^TM^ Post Primary (Leica Biosystems Newcastle, Newcastle upon Tyne, UK, RE7111)—to detect mouse antibodies, and subsequently incubation with Novolink™ Polymer occurred (Leica Biosystems Newcastle, Newcastle upon Tyne, UK, RE7112), a solution that recognizes the Post Primary and rabbit antibodies, and finally with DAB (3,3′-diaminobenzidine) Chromogen (Leica Biosystems Newcastle, Newcastle upon Tyne, UK, RE7105) diluted in the Novolink™ DAB Substrate Buffer (Leica Biosystems Newcastle, Newcastle upon Tyne, UK, RE7143). Sections were counterstained with Novocastra™ haematoxylin (Leica Biosystems Newcastle, Newcastle upon Tyne, UK, RE7107), dehydrated following standard procedures, and coverslipped with mounting medium (CV Mount, Leica Biosystems, REF 14046430011). Slides were evaluated using a ZEISS Observer Z1 light microscope (AxioVision 4.8 software; illumination system LUMEN 200; PRIOR, ×400 magnification) by two independent researchers (M.J.S. and K.P.). The sections were scored based on the percentage of positively stained tissue (P) for CNPase, Smad2, pSmad3, and TGFβ1 (<25% = 0; 25–75% = 1; >75% = 2). The level of staining intensity (I) was recorded as follows: 0—none, 1—weak, 2—moderate, 3—strong. Expression (E) of the staining was calculated by multiplying values of positivity (P) and intensity (I) for each section. The haematoxylin and eosin staining was performed on a few selected tissue sections from each group of patients. In the first step, tissues were deparaffinised, rehydrated, and stained with haematoxylin for 5 min. Afterwards, sections were rinsed with water, stained with eosin for 1.5 min, then dehydrated and coverslipped with mounting medium.

### 2.3. Immunofluorescence Tissue Staining

For immunofluorescence detection of TGFβ1 and CNPase expression, the same primary antibodies mentioned above were used. After standard deparaffinisation, rehydration, and antigen retrieval, sections were blocked in a 4% BSA-PBS solution (Sigma Aldrich, St. Louis, MO, USA, A7906) for 1.5 h at RT. Then, sections were incubated with the primary antibody or only with Antibody Diluent (negative controls) overnight at 4 °C in a moist chamber. After overnight incubation, tissues were incubated with a secondary antibody at RT in the dark. The following secondary antibodies were used: goat anti-mouse conjugated with Alexa 488 (1:1000, Life Technologies, Eugene, OR, USA, A-11001) and goat anti-rabbit conjugated with Alexa 594 (1:1000, Life Technologies, Eugene, OR, USA, A-11012). To counterstain the nucleus, sections were then incubated with DAPI solution (1:1000, Thermo Scientific, Rockford, IL, USA, 62248) and coverslipped with Fluorescent Mounting Medium (Dako, Carpinteria, CA, USA, S3023).

### 2.4. Statistical Analysis

Statistical analysis was performed using R programme language (v4.2.2) in the programme RStudio (v2023.03.0). The packages rstatix (v0.7.1) and Hmisc (v5.0-1) were used for analysis and ggpubr (v0.5.0) and corrplot (v0.92) for visualisation. The Kruskal–Wallis and Mann–Whitney tests were used to evaluate the differences between groups in selected immunohistochemistry and clinical parameters, and Spearman correlation analysis was used to assess the correlation between those factors.

## 3. Results

### 3.1. Levels of Smad2, pSmad3, TGFβ1, and CNPase Antigens in the Upper Airway Epithelium and Blood Eosinophils of CRSsNP, CRSwNP, and NC Patients

**Figure 1:**
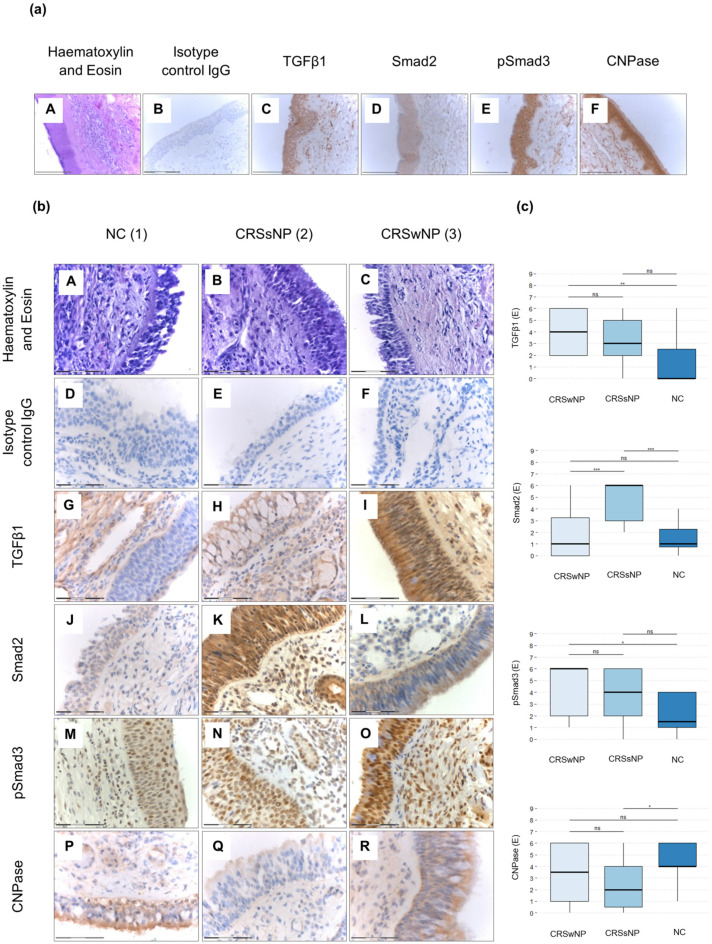
Tissue section staining. (**a**) Positive staining control tissue (normal skin): (**A**) H+E staining; (**B**) isotype control IgG; (**C**) TGFβ1; (**D**) Smad2; (**E**) pSmad3; (**F**) CNPase; (**b**) H+E; TGFβ1; Smad2; pSmad3; and CNPase immunohistochemical staining of the upper airway epithelium derived from normal control (NC) (1: **A**,**D**,**G**,**J**,**M**,**P**), CRSsNP (2: **B**,**E**,**H**,**K**,**N**,**Q**), and CRSwNP (3: **C**,**F**,**I**,**L**,**O**,**R**); 400× magnification; (**c**) Smad2, pSmad3, TGFβ1, and CNPase expression (E) in the upper airway epithelium of NC, CRSsNP, and CRSwNP (* *p* < 0.05, ** *p* < 0.01, *** *p* < 0.001, ns—non-statistical significance). The graphs present median values of staining expression.

**Figure 2:**
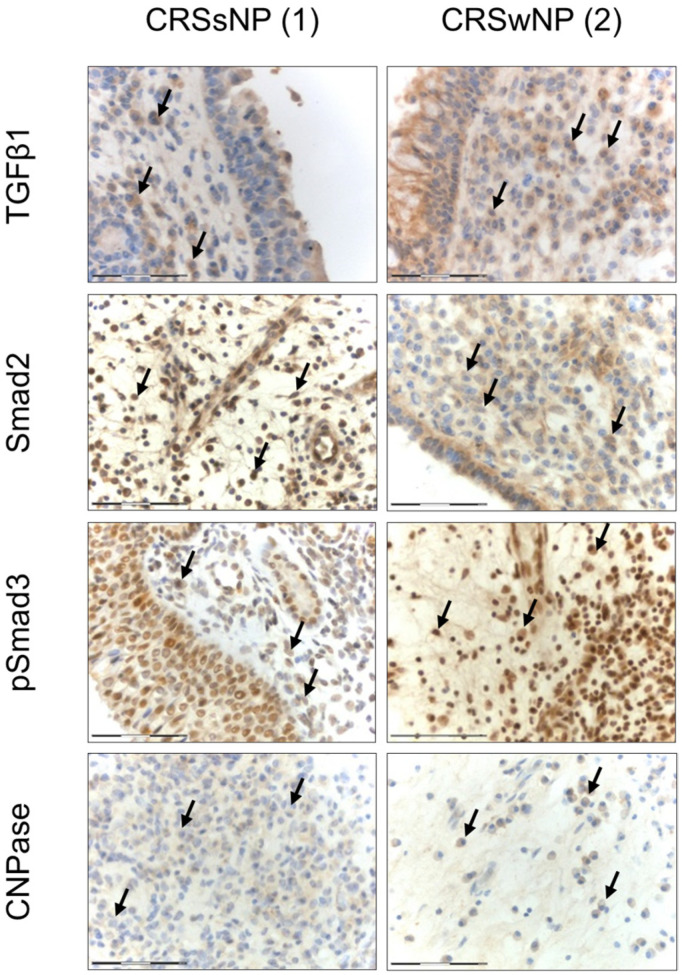
TGFβ1-, Smad2-, pSmad3-, and CNPase-positive cells (arrows) infiltrating the subepithelial area of the airway epithelium derived from CRSsNP (1) and CRSwNP (2) patients (400× magnification). The images present the representative TGFβ1+, Smad2+, pSmad3+, and CNPase+ cells in the subepithelial area that we have observed in the stained tissues; however, an evaluation of their quantity was not the aim of our current study.

Smad2-positive cells were detected in the UAE of all patient groups (Figure 1b(J–L)), with the highest levels of Smad2 expression observed in the CRSsNP patient group. Smad2 expression was significantly elevated in patients with CRSsNP compared to those with CRSwNP (*p* = 0.001). Furthermore, Smad2 expression was markedly higher in CRSsNP patients compared to NC patients (*p* < 0.001; Figure 1c). pSmad3-positive cells were present in the UAE of the CRSsNP, CRSwNP, and NC groups (Figure 1b(M–O)), with the highest expression levels of pSmad3 in the CRSwNP group. The expression of pSmad3 was significantly elevated in the CRSwNP group compared to the NC group (*p* = 0.007; Figure 1c). TGFβ1-positive cells were detected in UAE derived from the CRSsNP, CRSwNP, and NC groups (Figure 1b(G–I)), with expression significantly increased in CRSwNP patients. TGFβ1 expression was higher in CRSwNP patients compared to NC patients (*p* = 0.009; Figure 1c). Similarly, CNPase-positive cells were present in tissues from all three patient groups (Figure 1b(P–R)), with the highest levels in the NC group. CNPase expression was significantly decreased in CRSsNP compared to NC patients (*p* = 0.03; Figure 1c). Additionally, Smad2-, pSmad3-, TGFβ1-, and CNPase-positive cells were detected in the subepithelial matrix area in patients in the CRSsNP, CRSwNP, and NC groups (Figure 2).

**Figure 3:**
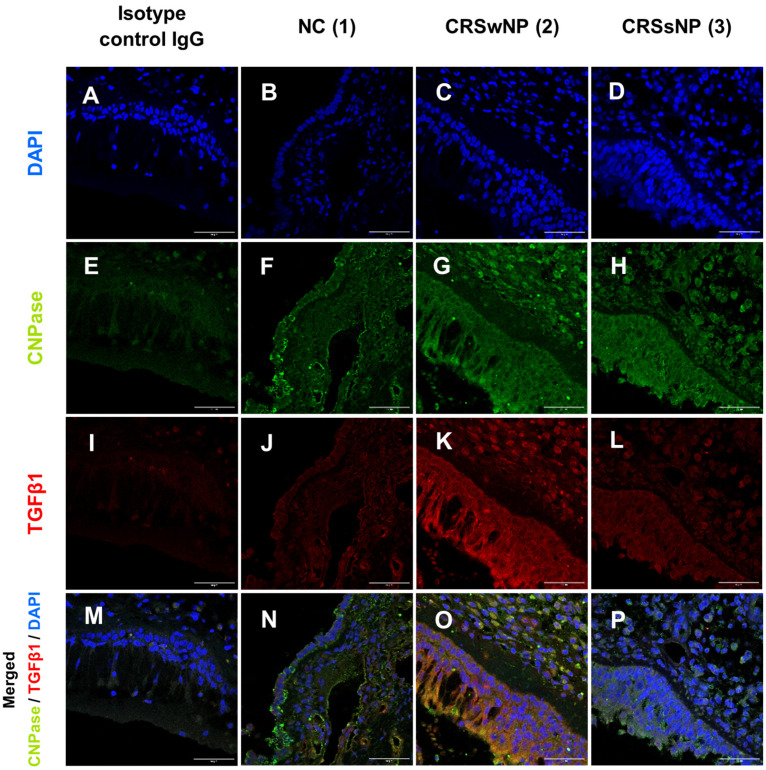
TGFβ1 and CNPase immunofluorescence staining of the upper airway epithelium derived from NC (1) (**B**,**F**,**J**,**N**), CRSwNP (2) (**C**,**G**,**K**,**O**), and CRSsNP (3) (**D**,**H**,**L**,**P**); Isotype control IgG (**A**,**E**,**I**,**M**); 600× magnification.

For the evaluation of co-expression, fluorescence staining was performed, revealing that TGFβ1 and CNPase were co-localised in the epithelium of patients with CRSsNP and CRSwNP, as well as in the epithelium harvested from NC patients (Figure 3).

**Figure 4:**
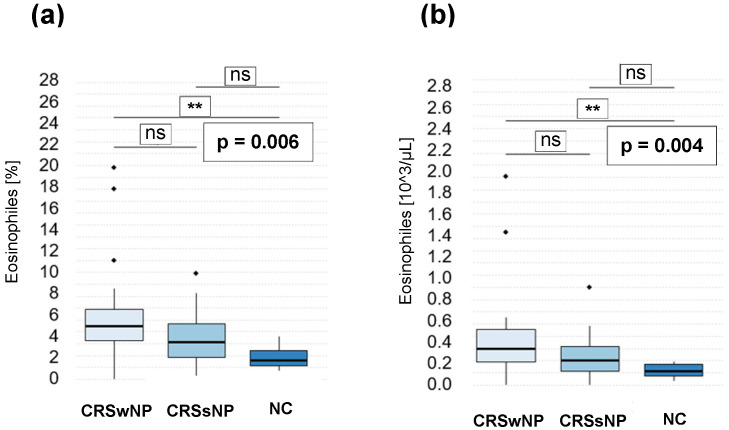
Eosinophiles in the blood of CRSwNP, CRSsNP, and NC at the percentage (**a**) and concentration [10^3^/μL] in the blood (**b**) (** *p* < 0.01); ns—non-statistical significance.

The levels of blood eosinophils were significantly elevated in patients with CRSwNP compared to NC, both in terms of their percentage in the blood (*p* = 0.006) and their absolute concentrations (*p* = 0.004; Figure 4a,b, respectively).

### 3.2. Expression Levels of Smad2, pSmad3, TGFβ1, and CNPase Correlate Positively with Each Other within Specific Patient Groups, Clinically Observed Symptoms, and Selected Questions from SNOT-20 and EPOS 2020 Questionnaires

**Figure 5:**
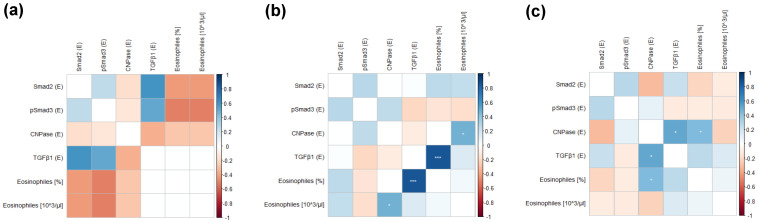
Correlation matrix between Smad2, pSmad3, TGFβ1, and CNPase immunostaining in the upper airway epithelium of NC (**a**), CRSwNP (**b**), and CRSsNP (**c**) and within groups (* *p* < 0.05, *** *p* < 0.001). Heatmaps present correlation between median values of antigen expression and quantities of blood eosinophiles.

The correlations between TGFβ1, Smad2, pSmad3, and CNPase antigens were assessed within NC, CRSwNP, and CRSsNP patients, and all relationships are presented in the correlation matrices in Figure 5a–c, respectively. In our study, groups did not differ by sex (Chi^2^ test).

#### 3.2.1. Correlations within Control Patients

**Figure 6:**
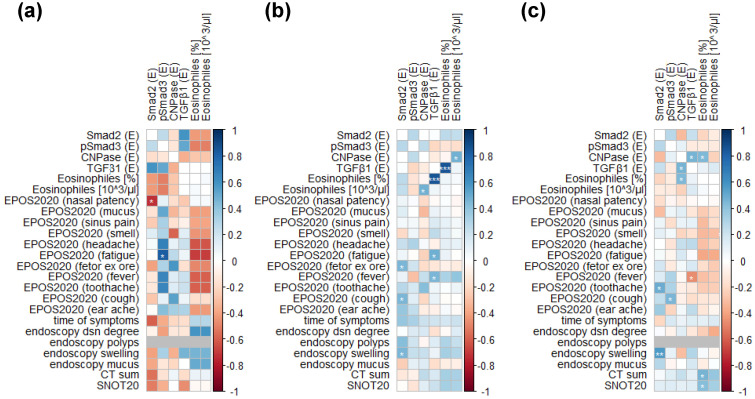
Correlation matrix between Smad2, pSmad3, TGFβ1, and CNPase immunostaining in the upper airway epithelium of NC (**a**), CRSwNP (**b**), and CRSsNP (**c**) and selected clinically observed symptoms (* *p* < 0.05, ** *p* < 0.01, *** *p* < 0.001). Grey cells represent parameters not applicable in the indicated group of patients.

**Figure 7:**
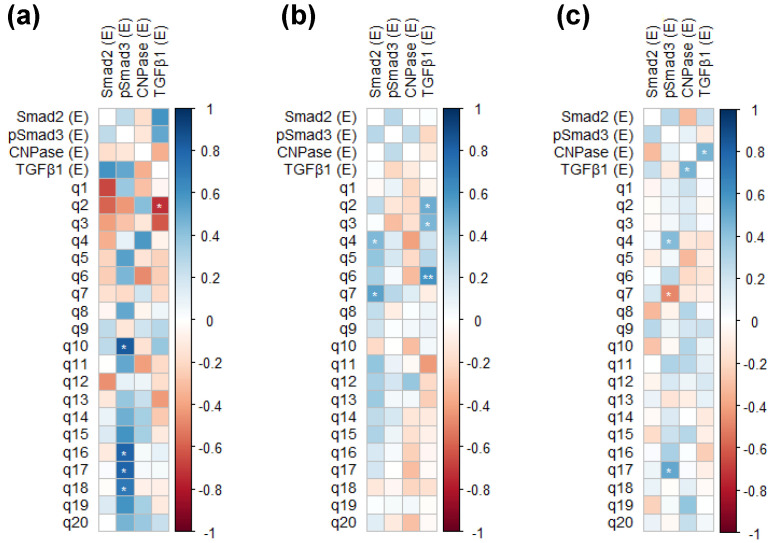
Correlation matrix between Smad2, pSmad3, TGFβ1, and CNPase immunostaining in the upper airway epithelium of NC (**a**), CRSwNP (**b**), and CRSsNP (**c**) and the SNOT-20 questions (* *p* < 0.05, ** *p* < 0.01). q 1—need to blow nose; q 2—sneezing; q 3—runny nose; q 4—cough; q 5—postnasal discharge; q 6—thick nasal discharge; q 7—ear fullness; q 8—dizziness; q 9—ear pain; q 10—facial pain/pressure; q 11—difficulty falling asleep; q 12—wake up at night; q 13—lack of good night’s sleep; q 14—wake up tired; q 15—fatigue; q 16—reduced productivity; q 17—reduced concentration; q 18—frustrated/restless/irritable; q 19—sad; q 20—embarrassed.

In NC patients, pSmad3 expression correlated positively with the following questions from the SNOT-20 questionnaire: q 10—facial pain (*p* = 0.01; r = 0.832), q 16—reduced productivity (*p* = 0.016; r = 0.805), q 17—reduced concentration (*p* = 0.017; r = 0,8), and with fatigue (*p* = 0.012; r = 0.826). Negative correlations were observed between the expression of Smad2 and nasal patency (*p* = 0.041; r = −0.726), as well as TGFβ1 with q 2—sneezing (*p* = 0.043; r = −0.0722) (Figure 6a and Figure 7a).

#### 3.2.2. Correlations within the Group of Patients with CRSsNP

In CRSsNP patients, positive correlations were observed between expression levels of CNPase and TGFβ1 (*p* = 0.049; r = 0.413). Additionally, in CRSsNP patients, CNPase expression correlated with the level of blood eosinophils (*p* = 0.0497; r = 0.414) (Figure 5c), as well as with patient age (*p* = 0.024; r = 0.472). In CRSsNP patients, pSmad3 expression correlated positively with the following questions from the SNOT-20 questionnaire (Figure 7c): q 4—cough (*p* = 0.036; r = 0.439), q 17—reduced concentration (*p* = 0.013; r = 0.512), and cough in the EPOS questionnaire (*p* = 0.028; r = 0.457). Negative correlations were found with q 7—ear fullness (*p* = 0.019; r = −0.483). Smad2 expression correlated positively with swelling observed in endoscopy (*p* = 0.005; r = 0.562) and toothache (*p* = 0.017; r = 0.490). TGFβ1 negatively correlated with fever (*p* = 0.025; r = −0.467) (Figure 6c and Figure 7c).

#### 3.2.3. Correlations within the Group of Patients with CRSwNP

In patients with CRSwNP, blood eosinophils positively correlated with the expression levels of CNPase (*p* = 0.012; r = 0.555) and TGFβ1 (*p* = 0.0000578; r = 0.749). TGFβ1 expression also showed positive correlations with fatigue (*p* = 0.032; r = 0.481), fever (*p* = 0.042; r = 0.458), q 2—sneezing (*p* = 0.027; r = 0.493), q 3—runny nose (*p* = 0.046; r = 0.451), and q 6—thick nasal discharge (*p* = 0.006; r = 0.594). Smad2 expression correlated positively with swelling observed in endoscopy (*p* = 0.045; r = 0.452), cough in the EPOS questionnaire (*p* = 0.041, r = 0.460), fetor ex ore (*p* = 0.043; r = 0.457), q 4—cough (*p* = 0.049; r = 0.446), and q 7—ear fullness (*p* = 0.016, r = 0.531) (Figure 6b and Figure 7b).

### 3.3. Age of Patients Correlated with the Subtype of CRS and CNPase Expression Level

The age of patients was significantly higher in the CRSwNP group compared to the CRSsNP group (*p* = 0.016) and NC group (*p* = 0.01). A positive correlation was observed between patient age and CNPase expression (*p* = 0.024) in CRSsNP patients.

## 4. Discussion

Current evidence indicates that tissue remodelling plays a crucial role in the pathogenesis of CRS [10,13,14,27]. It involves modifications in epithelial cells, resident fibroblasts, the ECM, and the activity and composition of infiltrating immune cells. These changes collectively contribute to oedema, fibrosis, pseudocysts [44], basement membrane thickening, goblet cell hyperplasia [18], submucosal gland alterations [45], and changes in vascularity [46]. Based on the literature, we hypothesised that TGFβ/Smad signalling and CNPase may play a role in tissue transformation in CRS.

In this study, we demonstrated the expression of TGFβ1, Smad2, pSmad3, and CNPase in the epithelium obtained from patients with CRSsNP and CRSwNP as well as NC, with several distinctions between patient groups. TGFβ1 expression was upregulated in the mucosa of CRSwNP compared to NC. Smad2 expression was significantly increased in the CRSsNP group compared to CRSwNP and NC. pSmad3 expression was overexpressed in CRSwNP compared to NC. In contrast, another study reported the increased expression of TGFβ1 and TGFβ2, a higher number of pSmad2-positive cells, and increased collagen expression in CRSsNP patients compared to controls, with the downregulation of TGFβ1, pSmad2, and collagen in CRSwNP patients [47]. Differences in these results may be due to variations in the tissues analysed [48], the type of sinuses, the disease state of patients, or differences in the technical approach to immunohistochemical staining and analysis.

The molecular mechanisms underlying TGFβ1/Smad2 activity in the UAE are not well understood. It has been shown that TGFβ1 treatment alters the morphology and activity of epithelial cells and fibroblasts. In bronchial epithelial cells, TGFβ1 triggers Smad3 phosphorylation and induces changes characteristic of EMT, including (1) the expression of cellular markers, (2) alterations in cellular morphology, (3) overexpression of proteins related to ECM and migratory capacities, and (4) upregulation of TGFβRI (TGF-β receptor I) and TGFβ1 itself [49]. Similarly, TGFβ1 induces EMT in epithelial cells derived from nasal polyps or the inferior turbinate, with this effect being particularly pronounced in tissues with high levels of TGFβ1 and Smad3 compared to healthy tissue samples [50], consistent with our findings. Fibroblasts residing in the subepithelial area of the upper airways are significant in CRS pathogenesis. TGFβ1 treatment activates Smad signalling, resulting in (1) the overexpression of EMT-related genes [51], (2) increased production of collagen and connective tissue growth factor (CTGF) [5], (3) enhanced migratory and contraction capacities [6], (4) formation of myofibroblasts, particularly in the pedicle area of nasal polyps, and (5) accumulation of fibronectin, which is significantly elevated in CRSwNP patients compared to controls [52,53,54]. We also observed a positive correlation between Smad2 expression and swelling observed during endoscopy in CRSsNP and CRSwNP patients, suggesting its role in tissue remodelling.

Our findings show enhanced Smad2 expression in CRSsNP patients. In renal epithelium, Smad2 inhibits Smad3 phosphorylation, nuclear translocation, and activation of target genes involved in ECM production and fibrosis [55]. Additionally, Smad7 can inhibit the translocation of the Smad2/3 complex to the nucleus, potentially regulated by Smad3 in a negative feedback loop [19,28]. Our data suggest that the interactions within the Smad family may differ between CRSsNP and CRSwNP patients.

In this study, we demonstrated for the first time, to the best of our knowledge, the expression of CNPase in the epithelium of CRSsNP, CRSwNP, and control non-CRS patients. The aim of this study was to evaluate the presence of CNPase in these tissues, compare its expression levels among the patient groups, and assess potential relationships with TGFβ1, Smad2, and pSmad3 proteins. The role of CNPase, particularly in non-myelinated cells, remains poorly understood and represents an exciting research gap with the potential to enhance our understanding of various disorders, including chronic upper inflammatory conditions. Based on the literature and our study, we propose several possible directions for CNPase activity. However, a detailed investigation of its mechanisms using advanced cell culture models and materials from a broader patient cohort is necessary.

Firstly, we found a positive correlation between CNPase and TGFβ1 expression in the sinus epithelium from CRSsNP patients, as well as their colocalisation in the epithelial area. Therefore, we hypothesize a potential synergistic effect of CNPase and TGFβ1 in promoting EMT. This hypothesis is supported by recent findings that CNPase promotes EMT in lens epithelial cells, leading to the increased expression of EMT-related markers (vimentin, α-SMA, and fibronectin). Interestingly, this process was also mediated by a member of the TGF family [35].

Secondly, CNPase expression was significantly decreased in the epithelium of CRSsNP patients compared to NC patients. However, this effect needs to be confirmed in a larger patient cohort to determine if it is a general characteristic of CRSsNP or specific to certain phenotypes. Additionally, including more non-CRS patients in the control group would be beneficial. The absence of CNPase enzyme activity in the tissue might lead to an accumulation of 2′,3′-cAMP, which is known to promote the opening of mitochondrial permeability transition pores (mPTPs), resulting in apoptosis, necrosis, and further tissue damage. In the case of kidney injury, CNPase has been shown to mediate the metabolism of 2′,3′-cAMP, leading to increased levels of ADO and an anti-inflammatory, renoprotective process [31]. Furthermore, cells lacking CNPase expression exhibit increased levels of secreted small extracellular vesicles [56], which play a role in regulating immune cell activities, tissue remodelling, and angiogenesis in CRS [54,57,58,59,60,61,62,63,64]. These vesicles are present in all body fluids and can transport proteins, lipids, and nucleic acids between neighbouring or distant cells/tissues, modulating their activities. For example, small extracellular vesicles isolated from the nasal mucosa of CRSwNP patients contained miR-375-3p, which inhibits a molecule involved in preventing EMT, thereby promoting EMT-triggering mechanisms [65]. Recent research has also found that the mitochondrial isoform of CNPase is crucial for inhibiting SARS-CoV-2 virion development [66]. Interestingly, a study of a Korean cohort demonstrated an increased risk of SARS-CoV-2 infection and severe COVID-19 in CRSsNP patients [67]. Moreover, in a murine model of viral acute rhinosinusitis, the topical intranasal administration of ADO reduced proinflammatory cytokines, cell damage, goblet cell hyperplasia, and mucus production, indicating an anti-inflammatory effect mediated through adenosine 2A (A_2A_) receptor signalling [68]. Additionally, exogenous aerosol containing ADO has been shown to enhance mucociliary clearance (MCC) [69].

In eosinophilic CRSwNP, increased serum concentrations of ADO are associated with tissue infiltration by eosinophils [70]. Additionally, serum levels of eotaxin in CRSwNP patients might predict postoperative recurrence [71]. Eosinophils infiltrating nasal polyps secrete TGFβ1, which leads to stromal fibrosis and basement membrane thickening [72]. In our study, we observed elevated levels of eosinophiles in the blood of CRSwNP patients compared to NC patients, as well as a positive correlation between blood eosinophile counts and tissue CNPase levels in both CRSwNP and CRSsNP patients, and with TGFβ1 in CRSwNP patients. Given the diminished levels of CNPase in the sinus epithelium of CRSsNP patients, the positive correlation between tissue CNPase and ADO in the bloodstream may have a potential diagnostic value.

Another possible aspect of decreased CNPase in the CRSsNP epithelium could be related to cilia dysfunction. MCC is a key defence mechanism of the nasal epithelium, helping to remove external factors such as environmental pollutants, allergens, and pathogenic microorganisms through mucous transport. In CRS patients, ciliary structure, beating, and function are often impaired [73,74,75], leading to mucous accumulation, an altered, infection-prone microenvironment, and hypoxic conditions. Interestingly, CNPase is associated with microtubules [41], which are crucial components of ciliary structure; thus, reduced CNPase expression might be linked to these dysfunctions.

There are some limitations to this study. The main limitation was the relatively small number of participants in the control group. We performed additional Cohen’s d effect size analysis (using rstatix v0.7.1 package for R) and power analysis (using pwr v1.3-0 package for R), showing six large effects, five small effects, and one moderate effect for tested molecules (Supplementary Table S2). Although the differences in CNPase expression between CRSwNP and NC patients were statistically insignificant, the highest scores in a few CRSwNP patients may reflect specific clinical states that could be more apparent in a larger cohort. We observed statistically significant differences in pSmad3 and TGFβ1 staining intensity between CRSwNP and CRSsNP patients, suggesting that larger studies may reveal statistically significant differences between these groups. Additionally, we noted the presence of TGFβ1-, Smad2-, pSmad3-, and CNPase-positive immune cells infiltrating the subepithelial area in tissues from CRSsNP, CRSwNP, and NC, indicating similar underlying mechanisms. Based on morphological characteristics, we assume that different cell populations are involved, including innate immune cells such as eosinophiles, basophiles, mast cells, and macrophages, as well as adaptive immune cells like T and B cells. Further staining for specific markers is needed to differentiate between these populations. Due to different eosinophil counts, there is a possibility that different endotypes were enclosed to the investigated groups of patients.

Another limitation is that the CRSwNP group was significantly older than the patients in the other two groups. However, this is consistent with other studies that have observed a correlation between nasal polyps and older age [76].

In this study, we identified correlations between selected antigens stained in the epithelium and common CRS symptoms, as assessed through the SNOT-20 and EPOS 2020 questionnaires and endoscopic examination. In CRSsNP, pSmad3, and CRSwNP, Smad2 correlated positively with cough. Cough reflex sensitivity might be mediated by nasal sensory nerves [77], which can be activated by viral and bacterial infections that also activate pSmad2 and pSmad3 [78]. Additionally, Smad2 and Smad3 may be involved in tissue remodelling, potentially irritating the nerves responsible for cough reflex. In CRSsNP, the positive correlation between pSmad3 and reduced concentration may result from impaired nasal airflow due to tissue remodelling, leading to sleep disturbances and cognitive issues. We also found a negative correlation between pSmad3 and ear fullness in CRSsNP patients. Larger patient cohorts have shown a 69% increased risk of developing middle ear cholesteatoma in CRS patients [79]. Cholesteatoma pathogenesis is associated with decreased Smad3 expression, which is suggested to have pro-apoptotic functions [80]. This protein may link CRS and cholesteatoma pathogenesis, though a further investigation is needed. In CRSsNP, Smad2 positively correlated with toothache, likely due to the close proximity of sinus cavities to the roots of the teeth, where both sinus inflammation and dental disorders may interact [81].

In CRSwNP, TGFβ1 positively correlated with sneezing, runny nose, and thick nasal discharge. TGFβ1 regulates mucin activity in the nasal epithelium [82], and mucins are the main components of airway mucus. The overexpression of gel-forming mucins MUC5AC and MUC5B can lead to symptoms such as runny nose or thick nasal discharge [83]. The activation of sensory nerves in the epithelium may also trigger sneezing. Smad2 positively correlated with ear fullness in CRSwNP. Otologic symptoms in CRS patients, such as Eustachian tube dysfunction (ETD), are prevalent between 15 and 42% [84] and can cause ear pain, pressure, and fullness. Mucosal oedema and sinus secretions may contribute to ETD by impairing the Eustachian tube’s ability to equalize pressure [85]. Therefore, Smad2′s involvement in tissue remodelling and immune cell activity might be related to these ear disorders. Smad2 also positively correlated with fetor ex ore in CRSwNP patients; however, the multifactorial nature of halitosis warrants further investigation into its relationship with Smad2.

We observed a correlation between TGFβ1 and fever, with CRSsNP showing a negative correlation and in CRSwNP a positive one. Since fever was excluded as a criterion at the time of surgery and was a general question in the questionnaire without specified duration, this represents a limitation of this study. To obtain conclusive results, a larger patient cohort is needed. Additionally, both groups showed a positive correlation between Smad2 expression and swelling observed during endoscopy, suggesting its critical role in tissue remodelling.

These findings indicate that TGFβ1, Smad2, and pSmad3 participate not only at the molecular level but also in the clinical symptoms of CRS. Their involvement in tissue remodelling and epithelial alterations may contribute to nasal blockage, obstruction, and reduced nasal airflow, which, as previously shown, can trigger sleep disturbances [86,87]. Although this study did not find a statistically significant direct correlation between TGFβ1 expression and sleep disorders, other analyses have reported associations between TGFβ1 and sleep disturbances in rabbit models [88] and CRS patients [89]. The data suggest that alterations in the TGFβ/Smad signalling pathway may reflect clinical symptoms and could be useful for diagnostic and therapeutic applications, but further detailed investigation and inclusion of a broader patient cohort are needed.

## 5. Conclusions

This study demonstrates the expression of TGFβ1, Smad2, pSmad3, and CNPase in the UAE obtained from CRSsNP, CRSwNP, and NC patients. To the best of our knowledge, this is the first study evaluating CNPase expression in the sinus epithelium, suggesting the existence of the 2′,3′-cAMP-ADO pathway in the upper airways. The identification of CNPase expression in the upper airway mucosa, along with its decreased levels in CRSsNP patients, sheds new light on the pathogenesis of respiratory inflammatory diseases and could lead to novel diagnostic and therapeutic approaches. The positive correlations between TGFβ1, Smad2, pSmad3, and CNPase in the sinus epithelium of CRS patients suggests the presence of unexplored synergistic pathways in disease pathogenesis that require further investigation.

## Figures and Tables

**Table 1:** Clinicopathological characterisation of CRSwNP, CRSsNP, and NC patients included in this study ^a^.

Characteristic	NC Patients (*n* = 8)	CRSwNP Patients (*n* = 20)	CRSsNP Patients (*n* = 23)
Sex			
Male	6	12	15
Female	2	8	8
Age			
Range	19–44	26–71	19–65
Median	32	48.5	35
Allergy	0	8	9
Asthma	1	3	2
Aspirin sensitivity/ non-exacerbated respiratory disease (N-ERD)	0	2	0
Eosinophiles [%] (average ± SD)	2.1 (±1.183)	6.118 (±4.94)	3.275 (±2.236)
Eosinophiles [10^3^/μL] (average ± SD)	0.129 (±0.063)	0.427 (±0.404)	0.229 (±0.178)

^a^ Statistical analysis for age: control vs. CRSsNP: *p* = 0.298; control vs. CRSwNP: *p* = 0.003; CRSwNP vs. CRSsNP: *p* = 0.003; for sex: control vs. CRSsNP: *p* = 0.944, control vs. CRSwNP: *p* = 0.755, CRSwNP vs. CRSsNP: *p* = 0.971.

## Data Availability

Data are contained within the article.

## Supplementary Materials

The following supporting information can be downloaded at: https://www.mdpi.com/article/10.3390/jpm14090894/s1. Table S1: Questionnaire based on the EPOS 2020 and fulfilled by patients in VAS scale (0–10; 0—not troublesome and 10—worst thinkable troublesome). Table S2: Results from Cohen’s d effect size and power analysis. (CI low and CI high—upper and lower confidence intervals; power—test power).
