# Supplementary material for: Evaluation of CNPase and TGFβ1/Smad Signalling Pathway Molecule Expression in Sinus Epithelial Tissues of Patients with Chronic Rhinosinusitis with (CRSwNP) and without Nasal Polyps (CRSsNP)
Source: J Pers Med. 2024 Aug 23;14(9):894. doi: 10.3390/jpm14090894 (PMC11433593; doi:10.3390/jpm14090894)
Supplement: Supplementary file 1 [file jpm-14-00894-s001.zip › Supplementary_Material_1.pdf]

Symptom	Assessment of severity on scale 0-10
Obstruction or nasal blockage	
Presence of nasal discharge or posterior/anterior nasal drip	
Facial pain or pressure	
Reduction or loss of smell	
Headache	
Fatigue	
Halitosis	
Fever	
Toothache	
Cough	
Pain or feeling of blockage and fullness in the ear	

Table S1. Questionnaire based on the EPOS 2020 and fulfilled by patients in VAS scale (0 -10; 0- not troublesome and 10 - worst thinkable troublesome).
