# Supplementary material for: Evaluation of CNPase and TGFβ1/Smad Signalling Pathway Molecule Expression in Sinus Epithelial Tissues of Patients with Chronic Rhinosinusitis with (CRSwNP) and without Nasal Polyps (CRSsNP)
Source: J Pers Med. 2024 Aug 23;14(9):894. doi: 10.3390/jpm14090894 (PMC11433593; doi:10.3390/jpm14090894)
Supplement: Supplementary file 1 [file jpm-14-00894-s001.zip › Supplementary_Material_2.pdf]

	.y.	group1	group2	Cohen's d effsize	particle	n1	n2	CI low	CI high	magnitude	power
**1**	result	Control	CRSsNP	-2.0384156	Smad2 (E)	8	23	-3.66	-1.16	large	0.9977163
**2**	result	Control	CRSwNP	-0.3683942	Smad2 (E)	8	20	-1.15	0.46	small	0.1356600
**3**	result	CRSsNP	CRSwNP	1.2258065	Smad2 (E)	23	20	0.60	2.24	large	0.9746352
**4**	result	Control	CRSsNP	-0.8395817	pSmad3 (E)	8	23	-1.88	-0.12	large	0.5070476
**5**	result	Control	CRSwNP	-1.2298378	pSmad3 (E)	8	20	-2.47	-0.45	large	0.8078005
**6**	result	CRSsNP	CRSwNP	-0.2611232	pSmad3 (E)	23	20	-0.94	0.33	small	0.1327319
**7**	result	Control	CRSsNP	0.9260954	CNPase (E)	8	23	0.19	1.99	large	0.5875351
**8**	result	Control	CRSwNP	0.4101513	CNPase (E)	8	20	-0.30	1.27	small	0.1567977
**9**	result	CRSsNP	CRSwNP	-0.4091545	CNPase (E)	23	20	-1.08	0.21	small	0.2574285
**10**	result	Control	CRSsNP	-0.7193492	TGFβ1 (E)	8	23	-2.18	0.15	moderate	0.3954619
**11**	result	Control	CRSwNP	-1.2346676	TGFβ1 (E)	8	20	-3.23	-0.26	large	0.8108187
**12**	result	CRSsNP	CRSwNP	-0.4612703	TGFβ1 (E)	23	20	-1.12	0.16	small	0.3135875

Table S2. Results from Cohen's d effect size and power analysis. (CI low and CI high - upper and lower confidence intervals; power – test power).
